# Oxidative Stress in Dilated Cardiomyopathy Caused by *MYBPC3* Mutation

**DOI:** 10.1155/2015/424751

**Published:** 2015-10-05

**Authors:** Thomas L. Lynch, Mayandi Sivaguru, Murugesan Velayutham, Arturo J. Cardounel, Michelle Michels, David Barefield, Suresh Govindan, Cristobal dos Remedios, Jolanda van der Velden, Sakthivel Sadayappan

**Affiliations:** ^1^Department of Cell and Molecular Physiology, Health Sciences Division, Loyola University Chicago, Maywood, IL 60153, USA; ^2^Institute for Genomic Biology, University of Illinois at Urbana-Champaign, Urbana, IL 61801, USA; ^3^Department of Cardiothoracic Surgery, University of Pittsburgh Medical Center, McGowan Institute for Regenerative Medicine, University of Pittsburgh, Pittsburgh, PA 15219, USA; ^4^Department of Cardiology, Thoraxcenter, Erasmus Medical Center, 's-Gravendijkwal 230, 3015 CE Rotterdam, Netherlands; ^5^Bosch Institute, Discipline of Anatomy and Histology, University of Sydney, Sydney, NSW 2006, Australia; ^6^Laboratory for Physiology, Institute for Cardiovascular Research, VU University Medical Center, van der Boechorststraat 7, 1081 BT Amsterdam, Netherlands

## Abstract

Cardiomyopathies can result from mutations in genes encoding sarcomere proteins including* MYBPC3*, which encodes cardiac myosin binding protein-C (cMyBP-C). However, whether oxidative stress is augmented due to contractile dysfunction and cardiomyocyte damage in* MYBPC3*-mutated cardiomyopathies has not been elucidated. To determine whether oxidative stress markers were elevated in* MYBPC3*-mutated cardiomyopathies, a previously characterized 3-month-old mouse model of dilated cardiomyopathy (DCM) expressing a homozygous* MYBPC3* mutation (cMyBP-C^(t/t)^) was used, compared to wild-type (WT) mice. Echocardiography confirmed decreased percentage of fractional shortening in DCM versus WT hearts. Histopathological analysis indicated a significant increase in myocardial disarray and fibrosis while the second harmonic generation imaging revealed disorganized sarcomeric structure and myocyte damage in DCM hearts when compared to WT hearts. Intriguingly, DCM mouse heart homogenates had decreased glutathione (GSH/GSSG) ratio and increased protein carbonyl and lipid malondialdehyde content compared to WT heart homogenates, consistent with elevated oxidative stress. Importantly, a similar result was observed in human cardiomyopathy heart homogenate samples. These results were further supported by reduced signals for mitochondrial semiquinone radicals and Fe-S clusters in DCM mouse hearts measured using electron paramagnetic resonance spectroscopy. In conclusion, we demonstrate elevated oxidative stress in* MYPBC3*-mutated DCM mice, which may exacerbate the development of heart failure.

## 1. Introduction

Heart failure (HF) remains a leading cause of morbidity and mortality worldwide and affects 5.1 million people in the United States [1]. Cardiomyopathies are a leading cause of HF and are still associated with substantial morbidity and mortality despite optimal treatment [2]. Therefore, novel therapeutic targets and treatments are required. Over the past 20 years, many inherited mutations within genes encoding proteins of the cardiac sarcomere have been identified as causing dilated (DCM) and hypertrophic (HCM) cardiomyopathy [2]. The genetic basis of HCM has been well established with more than 1400 mutations in genes encoding sarcomeric proteins having been identified [3, 4]. For example, mutations in* MYBPC3*, which encodes the contractile regulatory protein cardiac myosin binding protein-C (cMyBP-C), are the most common cause of HCM accounting for approximately 40% of the identified mutations [5]. Furthermore, it is estimated that DCM is triggered by mutations in sarcomeric protein genes, including* MYPBC3*, in 35–40% of genetic cases [6]. Due to sarcomere protein mutations, these cardiomyopathies are typically characterized by severe contractile dysfunction that worsens over time promoting either cardiac hypertrophy or dilation, depending on the phenotypic response of the heart to the mutation [7]. These responses involve progressive myocardial remodeling that includes myocyte disarray and damage that may lead to increased cardiac oxidative stress.

A previous study by Marian et al., using a genetic mouse model of HCM caused by mutation of cardiac troponin T (cTnT), examined the pathogenesis of interstitial fibrosis in terms of the balance between oxidants and antioxidants regulating normal collagen homeostasis [8]. Their findings indicated that antioxidant therapy leads to reduced myocardial oxidative stress and fibrosis [8]. Lombardi et al. later confirmed this in *β*-myosin heavy-chain transgenic rabbits, which had reversed cardiac myocyte hypertrophy and interstitial fibrosis with a reduction in oxidized to total glutathione ratio upon antioxidant therapy, indicating the potential benefit of antioxidant therapy in the treatment of human HCM [9]. Recently, Lu et al. have demonstrated that cardiac oxidative stress is elevated in the cTnT^R141W^ mouse model of DCM, which is inhibited by knockdown of CYP2E1 leading to an improved cardiac phenotype [10]. However, whether oxidative stress results from tissue damage caused by contractile dysfunction and myocardial remodeling in sarcomere protein-mutated forms of cardiomyopathy other than by cTnT mutation requires further elucidation.

In this study, we use a well-characterized mouse model of genetic DCM (cMyBP-C^(t/t)^) having a homozygous* MYBPC3* sarcomere gene mutation that produces truncated cMyBP-C variant [11–14] and heart tissue from human cardiomyopathy patients for comparison. We tested whether oxidative stress is elevated in cardiomyopathies caused by* MYBPC3* mutation and if this corresponds to worsened cardiac function and increased myocyte damage. Our data extend previous findings in models of HCM and DCM to include DCM caused by* MYBPC3 *mutations as having elevated oxidative stress that corresponded to severe cardiac dysfunction, myocyte damage, and myocardial remodeling.

## 2. Materials and Methods

### 2.1. Patients

For human studies, the investigation conformed to the principles outlined in the Declaration of Helsinki. The IRB at Loyola University Chicago approved the present study using the deidentified human heart tissue samples (LU number 205840). Human heart samples used here were from the Sydney Heart Bank and were collected under HREC approvals from the University of Sydney (HREC number 2814) and from St. Vincent's Hospital (HREC number H91/048/1a). The cardiomyopathy patient group consisted of 10 men and 4 women. Mean patient age was 43.3 ± 14.1 years. The donor group consisted of 9 men and 4 women with no known cardiovascular disease. Mean patient age was 43.5 ± 12.3 years. Detailed patient clinical characteristics are provided in Table 1.

### 2.2. Mouse Model of DCM

A knock-in mouse model of DCM [11], in which a homozygous mutation in* MYBPC3* causes a C′-modified cMyBP-C (cMyBP-C^(t/t)^) that does not incorporate into sarcomeres resulting in a cMyBP-C null heart [11], and age matched wild-type (WT) mice (FVB/N) were used to determine the association between oxidative stress, cardiac function, and myocardial remodeling in sarcomere protein-mutated DCM. Male and female WT and cMyBP-C^(t/t)^ animals were used for these studies. All animal protocols were approved by the Institutional Animal Care and Use Committee at Loyola University Chicago (LU number 205109) and were in accordance with the guidelines listed in the* Guide for the Use and Care of Laboratory Animals* published by the National Institutes of Health. Prior to organ harvesting, animals were euthanized in a carbon dioxide (CO_2_) chamber by slow flow of CO_2_ (10–30% of chamber volume per minute) followed by continued exposure for 15–30 minutes after breathing had stopped.

### 2.3. *In Vivo* Cardiac Function by Noninvasive Echocardiography

Cardiac function in WT and DCM mice that were anesthetized with 2% Isoflurane via inhalation was assessed by echocardiography using a Vevo 2100 (Fujifilm, Visual Sonics, Inc., Toronto, Canada) with a MS-550D 22–55 MHz transducer. Left ventricular internal diameter, wall thickness, and contractile function were measured using parasternal long-axis M-mode imaging and the Visual Sonics Vevo 2100 analysis package as described previously [12].

### 2.4. Histopathological Analyses

To assess the gross morphology of DCM versus WT hearts, the hearts were removed from anesthetized mice and were then drained of blood and fixed in 10% formalin. Hearts were bisected longitudinally or cross-sectioned at the base and dehydrated with a graded series of alcohols before being embedded in paraffin. Then, 5 *μ*m step-serial sections were taken from WT and DCM hearts and stained with hematoxylin and eosin (H&E) or Masson's trichrome. The H&E and Masson's trichrome-stained sections were imaged using the slide scanning system (Nanozoomer RT 2.0, Hamamatsu Corporation, Tokyo, Japan) using a 20x Uplansapo objective at a resolution of 0.23 *µ*m under bright field mode. To analyze the cellularity (nuclear area) and fibrosis, high-resolution images were exported from whole heart scanned sections. Images were thresholded based on RGB values for either nuclei (using the H&E labeled sections) or fibrosis (using trichrome-labeled sections) and the area of pixels occupied by these components was quantified in the program Axiovision (version 4.8, Carl Zeiss Microimaging, Jena, Germany).

### 2.5. Biochemical Measurements of GSH/GSSG Ratio

The ratio of reduced GSH to oxidized GSH (GSSG) serves as a marker of oxidative stress and redox status [15, 16]. The GSH/GSSG ratio was measured in WT and DCM heart homogenates as well as donor and cardiomyopathy human heart tissue homogenates using Cayman's GSH assay kit (Cayman Chemical, Cat. number 703002) as per the manufacturer's instructions. To prepare tissue homogenate, isolated hearts from WT and cMyBP-C^(t/t)^ mice were perfused with PBS (pH 7.4) prior to dissection to remove red blood cells and clots and a freeze clamp was used to freeze the tissue. Heart tissue from WT and DCM mice, as well as from donor and cardiomyopathy patients, was homogenized in cold buffer (phosphate, pH 6-7, and 1 mM EDTA) and centrifuged at 10,000 ×g for 15 minutes at 4°C. Samples were deproteinated with equal volumes of metaphosphoric acid (MPA, Sigma-Aldrich number 239275), vortexed, allowed to stand at room temperature for 5 minutes, and centrifuged at >2,000 g for 2 minutes. Supernatant was collected and stored at −20°C. To assay total GSH (oxidized and reduced forms), 4 M triethanolamine (TEAM, Sigma-Aldrich number T58300) was added to the supernatant, which was then vortexed. To quantify GSSG independent of GSH, 1 M 2-vinylpyridine (Sigma-Aldrich number 13229-2) in ethanol was added to deproteinated serum. Samples were mixed and incubated at room temperature for 60 minutes. Total GSH or GSSG levels were determined by adding the samples into wells of a 96-well plate. An assay cocktail containing MES buffer, reconstituted Cofactor Mixture, reconstituted Enzyme Mixture, water, and reconstituted DTNB was added to the sample wells. The plate was covered and incubated in the dark on an orbital shaker. Absorbance in the wells was read at 405 nm at 5-minute intervals for 30 minutes using the BioTek Epoch microplate reader. The data were analyzed using the Gen5 5.1.11 Data Analysis Software (BioTek).

### 2.6. Protein Carbonyl Assay

Carbonyl groups (aldehydes and ketones) are produced from the oxidation of protein side chains [17]. They react with 2,4-dinitrophenylhydrazine (DNPH) generating protein-bound 2,4-dinitrophenylhydrazones, which can be quantitated spectrophotometrically [18]. Therefore, protein carbonyl content serves as a general indicator of protein oxidation, oxidative stress, and oxidative damage [17, 18]. Protein carbonyl content was determined in WT and DCM heart tissue homogenates and in donor and cardiomyopathy human heart tissue homogenates using Cayman's Protein Carbonyl Colorimetric Assay Kit (Cayman Chemical, Cat. number 10005020) as per the manufacturer's instructions. Tissue was homogenized in cold buffer (50 mM MES or phosphate buffer pH 6.7 containing 1 mM EDTA) and centrifuged at 10,000 ×g for 15 min at 4°C. Sample and control tubes were prepared by adding 200 *μ*L of supernatants from centrifuged tissue samples into two tubes (sample and control) followed by addition of 800 *μ*L of 2,4-dinitrophenylhydrazine (DNPH) to the sample tube and 800 *μ*L of 2.5 M HCL to the control tube. Both tubes were incubated in the dark for 60 min at room temperature with 15 min intermittent vortexing. Then, 1 mL of 20% trichloroacetic acid (TCA) was added to each of the tubes, which were vortexed and incubated on ice for 5 min. Samples were centrifuged at 10,000 ×g for 10 minutes at 4°C and the supernatant was discarded. Pellets were resuspended in 10% TCA and incubated on ice for 5 min. The samples were centrifuged as above and the supernatants were discarded. The pellets were washed with 1 mL of a 1 : 1 ethanol : ethyl acetate mixture. Then, the pellets were resuspended, vortexed, and centrifuged as above. The washing procedure was performed two more times. Finally, the pellets were resuspended in 500 *μ*L guanidine hydrochloride by vortexing and were centrifuged as above to remove debris. The amount of protein-hydrazone produced was quantified spectrophotometrically at 360 nm. The carbonyl content was then normalized to protein concentration.

### 2.7. Lipid Peroxidation Assay

The oxidation of polyunsaturated fatty acids generates reactive carbonyl compounds including the aldehydic compounds malondialdehyde (MDA) and 4-hydroxyalkenal (HAE), which have been used as markers of lipid peroxidation and oxidative damage [19, 20]. MDA + HAE content was measured in WT and DCM mouse heart tissue homogenates and in donor and cardiomyopathy human heart tissue homogenates by a lipid peroxidation colorimetric assay (Oxford Biochemical Research, Product number FR 12) as per the manufacturer's instructions. Tissues were homogenized in 20 mM potassium phosphate buffer (pH 7.4) containing 0.5 M butylated hydroxytoluene (W218405-1KG-K Sigma). Lysate was clarified by centrifugation at 3000 ×g at 4°C and supernatant was collected in a fresh tube. Protein concentration was measured using Coomassie-Plus (Bradford) protein assay (Cat. number 23236 Thermo Scientific Rockford, IL). The assay was performed by taking 200 *μ*L of lysate mixed with 650 *μ*L of diluted R1 reagent (N-methyl-2-phenylindole in acetonitrile). Samples were gently mixed by vortexing followed by addition of 150 *μ*L of R2 reagent (methanesulfonic acid [MSA]). Samples were mixed well and tubes were stoppered and then incubated at 45°C for 60 min. Turbid samples were centrifuged at 15,000 ×g for 10 min to obtain a clear supernatant. Absorbance at 586 nm was measured for the supernatant along with the MDA standards (1,1,3,3-tetramethoxypropane [TMOP] in Tris-HCl). Separate sample blanks and reagent blanks were used to subtract the background. The concentration of analyte in each unknown was calculated from the net *A*_586_ of the sample using the following formula:

(1) $$\begin{array}{c} \left[\;MDA\;\right]=\frac{A_{586}-b}{a}\cdot df, \end{array}$$

where [MDA] is the *μ*M concentration of MDA in the sample, *A*_586_ = net absorbance at 586 nm of the sample, *a* = regression coefficient (slope), *b* = intercept, and df = dilution factor.

### 2.8. Electron Paramagnetic Resonance (EPR) Spectroscopy

EPR spectroscopic analysis was used to measure the free radical and paramagnetic species. The excised heart tissues were flash frozen and stored in liquid nitrogen for EPR spectroscopic analysis to measure the free radical and paramagnetic species. Each EPR sample was prepared by transferring the frozen heart tissue (160–270 mg) into a ceramic mortar prechilled with liquid nitrogen [21]. The tissue was then broken into small pieces in liquid nitrogen using a pestle. The tissue was then loaded into a finger Dewar containing liquid nitrogen. Low temperature, 77 K, and EPR spectra were recorded with a Bruker ESP 300E spectrometer (Bruker BioSciences, Billerica, MA, USA) operating at X-band with 100 kHz modulation frequency and a TM_110_ cavity as described previously [21]. The finger Dewar containing heart tissue samples in liquid nitrogen was placed within the EPR spectrometer cavity. All spectra were recorded with the following parameters: microwave frequency = 9.45 GHz, modulation amplitude = 5 G (2 G for semiquinone radical signals), time constant = 164 ms, scan time = 60 s, microwave power = 20 mW (1 mW for semiquinone radicals), and number of scans = 10. After this experiment, the heart tissue samples were used for the measurement of reactive oxygen species (ROS) formation. The EPR spin probe 1-hydroxy-3-carboxymethyl-2,2,5,5-tetramethyl-pyrrolidine (CMH, EPR silent) is oxidized by ROS and peroxynitrite (OONO^−^) to 3-carboxymethyl-2,2,5,5-tetramethyl-pyrrolidinyloxy radical (CM^∙^, EPR active) [22]. Heart tissues (50 mg) were treated with spin probe CMH (Alexis Biochemicals/Enzo Life Sciences) (1 mM) in Krebs buffer containing deferoxamine mesylate (Sigma-Aldrich) (25 *μ*M) and incubated at 37°C in a water bath for 30 minutes. The supernatant was collected and used for the CM^∙^ analysis. Samples were loaded into capillary tubes and EPR spectra were recorded at room temperature using a Bruker X-band EMX premiumX spectrometer. The following acquisition parameters were used: microwave power, 20 mW; modulation frequency, 100 kHz; modulation amplitude, 0.5 G; time constant, 82 ms; scan time, 41 s; and number of scans, 1.

### 2.9. Second Harmonic Generation Imaging (SHG) of Sarcomere Pattern and FFT Analysis

SHG imaging of myosin complex in representative WT and DCM hearts was accomplished and quantified as described previously [23–25]. The myosin complex was imaged without any labeling using the scattering process of sarcomere in forward directed SHG signals and the fibrous collagen was picked up by the backward directed SHG signals [25] using the H&E labeled whole hearts. All imaging, including the autofluorescence of tissue sections together with SHG signals, was performed using the LSM 710 confocal microscope equipped with nondescanned detectors. The excitation was 780 nm by two-photon laser and the detection bands are 380–400 nm as the scatters are exactly twice the frequency of excitation. The autofluorescence of the tissues was detected simultaneously between 600 and 650 nm [25]. The integrity of sarcomere pattern was analyzed using the Fast Fourier Transformation analysis in the program Fiji [26]. In addition, the loss of sarcomere pattern was analyzed using line intensity profiles measurement, which reveals fine structure by showing peaks whenever the fine structure is present. If not, these intensity profiles were devoid of peaks (flat lines). These intensity profiles are made using the aforementioned Axiovision program (version 4.8, Carl Zeiss Microimaging, Jena, Germany).

### 2.10. Statistical Analysis

Data are represented as mean values ± SEM. Significant differences between mean values were determined using Student's *t*-test. Statistically significant differences were considered as *P* < 0.05.

## 3. Results

### 3.1. DCM Hearts Display Contractile Dysfunction, Fibrosis, and Myocardial Disarray

**Figure 1:**
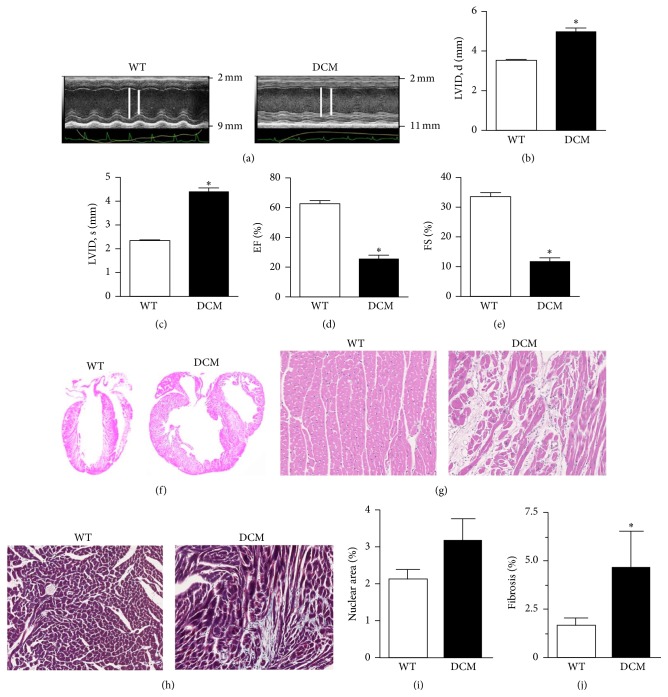
DCM hearts display contractile dysfunction, fibrosis, and myocardial disarray. (a) Representative parasternal long-axis M-mode echocardiographic images of WT and DCM hearts. Left ventricular internal diameter (LVID) is depicted by white lines (*note*: difference in scale between images). LVID at (b) peak diastole and (c) peak systole in WT and DCM hearts (*n* = 9 hearts, ^*∗*^ *P* < 0.001). Functional measurements derived from LVID measurements showing (d) ejection fraction (EF) and (e) fractional shortening (FS) in WT and DCM hearts (*n* = 9 hearts, ^*∗*^ *P* < 0.001). (f) H&E labeled whole hearts from WT and DCM animals. (g) H&E or (h) Masson's trichrome-labeled regions of interest (ROI) from WT and DCM hearts. (i) Quantification of cellularity based on nuclear area between WT (*n* = 5 hearts, 5 sections per heart) and DCM hearts (*n* = 4 hearts, 5 sections per heart). (j) Quantification of fibrosis (trichrome staining) area between WT (*n* = 4 hearts, 20 sections per heart) and DCM hearts (*n* = 4 hearts, 20 sections per heart; ^*∗*^ *P* < 0.0225). Data are represented as mean ± SEM. Significant differences (*P* < 0.05) between mean values were determined using Student's *t*-test.

Hearts from the cMyBP-C^(t/t)^ genetic mouse model of DCM have previously been characterized as having increased LV diastolic and systolic diameter and reduced LV fractional shortening (FS) suggestive of a DCM phenotype [11]. To confirm the presence of DCM in the cMyBP-C^(t/t)^ mice used here, we implemented parasternal long-axis M-mode echocardiography and assessed cardiac function in these animals compared to WT animals (Figure 1(a)). DCM animals had a significant elevation in LVID at both peak diastole (Figure 1(b)) and peak systole (Figure 1(c)) indicating dilation of the LV chamber. No significant difference was observed in total wall thickness in the hearts of WT and DCM animals (data not shown). However, we found a significant reduction in ejection fraction (EF) (Figure 1(d)) and FS (Figure 1(e)) in DCM hearts compared to WT hearts signifying impaired cardiac function in DCM hearts.

McConnell et al. previously characterized homozygous cMyBP-C^(t/t)^ DCM heart tissue as having prominent histologic abnormalities [11]. Here, we performed histopathological evaluation of WT and DCM hearts using H&E and Masson's trichrome staining. H&E staining of WT and DCM whole hearts revealed robust dilation of DCM hearts from cMyBP-C^(t/t)^ animals (Figure 1(f)). H&E staining of WT and DCM myocardial sections indicated eccentric hypertrophy and myocardial disarray in DCM hearts (Figure 1(g)). We observed a higher tendency of cellularity in DCM hearts quantified from H&E labeled sections (Figure 1(i)). The level of fibrosis captured (Figure 1(h)) and quantified (Figure 1(j)) by Masson's trichrome staining of myocardial sections from WT and DCM hearts revealed significantly elevated fibrosis in hearts from DCM animals compared to WT hearts. These data indicate scar tissue development, possible cell invasion upon cardiac remodeling, and loss of structural integrity in DCM hearts.

### 3.2. SHG Imaging of DCM Hearts Depicts Loss of Cellular Structure and Myocyte Damage

**Figure 2:**
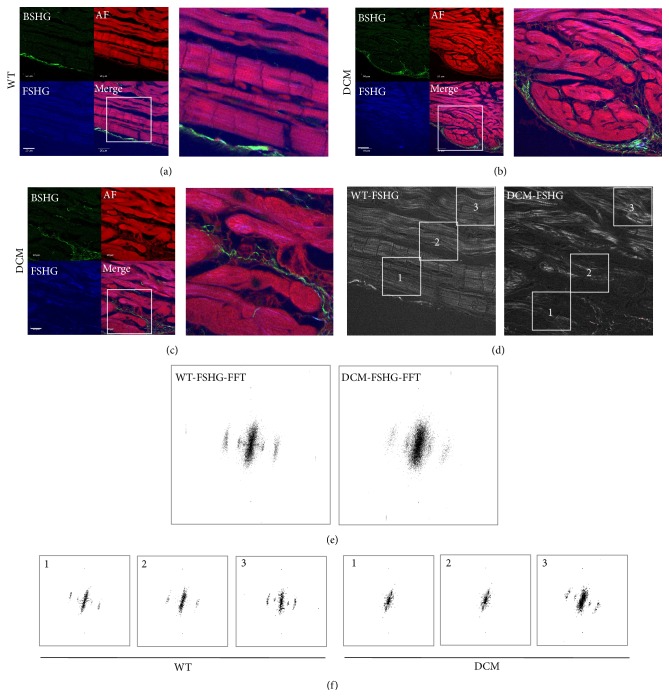
Second harmonic generation (SHG) imaging demonstrates disorganized sarcomere pattern in DCM hearts. (a) The sarcomere pattern in WT hearts closer to the inner chamber is discernible, is well-organized, and has low levels of collagen (green) (scale bar, 133 *μ*m). (b) In contrast, DCM hearts lack an organized sarcomere pattern (scale bar, 50 *μ*m). (c) High magnification (HM) image of an area in (b), showing clear loss of sarcomeric structure in these swollen areas. Green channel is backward SHG (BSHG) depicting predominantly collagen fibers, red channel is autofluorescence (AF), and blue channel is forward directed SHG (FSHG) showing sarcomere pattern and their merge. (d) Fast Fourier Transformation (FFT) analysis of the sarcomere pattern in WT and DCM hearts from the inner chamber area to distal parts of the hearts. (e) FFT of the whole frames in (d), note the fuzziness in the high frequency pattern. However, the localized changes in sarcomere pattern could not be discerned while doing whole frame FFT analysis. (f) FFTs of regions of interest (1, 2, and 3) from the inner chamber of heart to distal part as depicted in highlighted areas in (d) showing the region specific disorganization in sarcomeric pattern. Note there is no difference in FFT pattern in location 3 of both WT and DCM hearts, although, in regions closer to the inner chamber (2) and especially in swollen regions (1), there is a substantial difference in DCM hearts but there are no discernible differences in WT. Images ((a)–(d)) are representative of multiple locations of 2 hearts (*n* = 2).

**Figure 3:**
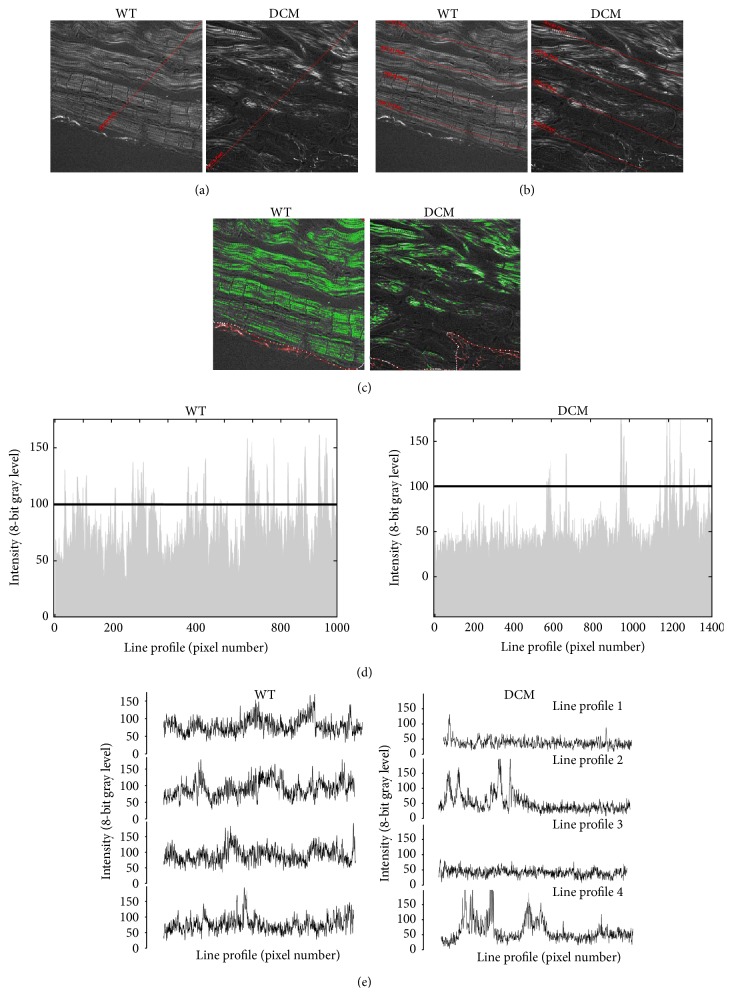
Loss of high frequency information (bands) directly indicates loss of typical sarcomere ladder structure in DCM hearts. Sarcomere fine structure in images from Figure 2(d) for DCM and WT heart sections is traced by drawing line intensity profiles across the cellular structures. (a) Loss of cellular structure in DCM (right) compared to WT (left) heart sections is indicated by drawing a single line from the inner chamber, which is (d) represented by line intensity profiles for both WT (left) and DCM (right) sections. (b) Loss of cellular structure is further indicated by multiple line profiles drawn left to right in DCM (right) compared to WT (left) heart sections, which is (e) represented by line intensity profiles (1–4) for both WT (left) and DCM (right) sections. (c) Sarcomere area imaging in WT (left) and DCM (right) hearts.

Diminished fractional shortening in DCM hearts suggested dysfunction at the level of the sarcomere. Previous methods looking at sarcomere structure used electron microscopy [10], limiting the cellular area that could be imaged and preventing the observation of other nearby defects. Therefore, we used second harmonic generation (SHG) imaging of WT and DCM heart tissue sections to simultaneously detect cellular structure and collagen deposition. Compared to WT hearts (Figure 2(a)), the SHG analysis indicated clear disorganization of cellular structure in DCM hearts (Figures 2(b) and 2(c)), especially at sites closer to the inner chamber wall where myocytes were most damaged. WT hearts showed well-organized sarcomere patterns in areas spanning from close to distant from the inner wall of the chamber (Figure 2(d),* left*). Sites radially away from the inner chamber were not disorganized to the extent as near the chamber in DCM hearts (Figure 2(d),* right*). This pattern of disorganization was revealed by the forward SHG images while backward SHG images depicted increased collagen presence (compare green channel in WT and DCM images), indicative of fibrosis. In accordance with this, the Fast Fourier Transformation (FFT) analysis of sarcomere pattern integrity of the images at the sites of damage confirmed the above inference. The disorganization was much higher at subset areas closer to inner chamber where the high frequency bands are completely lost in DCM hearts (Figures 2(e),* right*, and 2(f),* right*) compared to WT hearts (Figures 2(e),* left*, and 2(f),* left*). Loss of sarcomere pattern is quantified further using line intensity profiles. Profiles with fewer spikes indicate loss of fine structure (flat profiles). Line intensity profiles of DCM hearts produced fewer spikes compared to the WT hearts. This was irrespective of whether a single line was drawn across the entire image (Figure 3(a)) as depicted by radial line intensity profiles (Figure 3(d)), or if multiple line profiles were drawn on cell rows (Figure 3(b),* left* to* right*) starting from the chamber to cell rows away from the chamber (line intensity profiles in Figure 3(e), profiles 1–4). Furthermore, DCM hearts had 52% lower sarcomere area than WT hearts (Figure 3(c)). Together, these data are indicative of severe cardiac myocyte damage in DCM hearts.

### 3.3. Increased Oxidative Stress in Hearts from DCM Animals and Cardiomyopathy Patients

**Figure 4:**
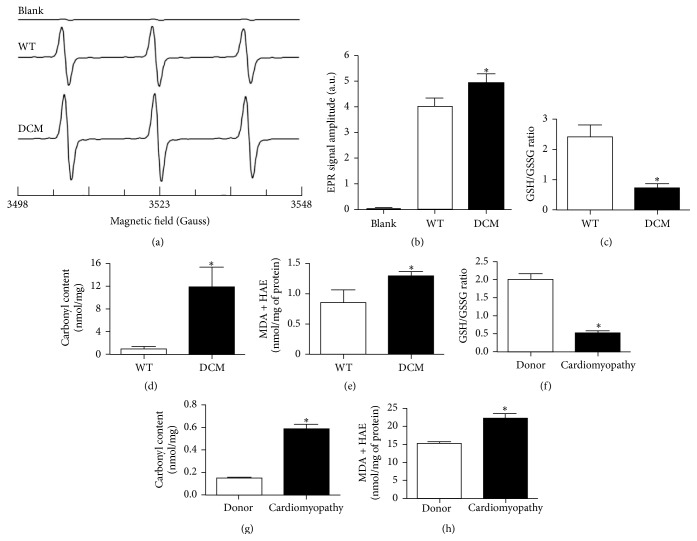
Increased oxidative stress in the hearts of DCM animals and cardiomyopathy patients. (a) Representative EPR spectra of CM^∙^ measured at room temperature. Heart tissues (50 mg) were treated with CMH (1 mM) in Krebs buffer containing deferoxamine mesylate (25 *μ*M) and incubated at 37°C in a water bath for 30 minutes. The reactive oxygen species oxidize CMH into CM^∙^. Blank samples are in the absence of heart tissue. (b) Summary data of CM^∙^ (*n* = 3 hearts, ^*∗*^ *P* < 0.034 in DCM versus WT by Student's *t*-test, and blank shown for representation). No significant accumulation of CM^∙^ was observed in the absence of heart tissues. (c) Comparison of GSH/GSSG ratios (*n* = 6 hearts), (d) carbonyl content (*n* = 6 hearts), and (e) MDA and HAE content (*n* = 8 hearts) in WT and DCM mouse heart tissue homogenates (^*∗*^ *P* < 0.05). (f) Comparison of GSH/GSSG ratios, (g) carbonyl content, and (h) MDA and HAE content in donor (*n* = 13 hearts) and cardiomyopathy (*n* = 14 hearts) human heart tissue homogenates (^*∗*^ *P* < 0.001). Data are represented as mean ± SEM. Significant differences (*P* < 0.05) between mean values were determined using Student's *t*-test.

Mutations in sarcomeric proteins often generate inefficiency in contraction that cannot cope with the demand required for proper contractile function leading to severe mechanical stress. Over time, progressive deterioration of contractile function perpetuates myocyte damage (Figures 2 and 3), which may stimulate oxidative stress that aggravates the progression of cardiomyopathies caused by sarcomere protein mutation. Indeed, oxidative stress has been demonstrated to be elevated in human HCM [27] as well as DCM caused by viral infection, inflammation, toxic substances [28], and mutation of cTnT [10]. As we observed severe cardiac dysfunction, fibrosis, and myocyte damage, we next determined whether oxidative stress was elevated in the hearts of cMyBP-C^(t/t)^ DCM mice. ROS formation was significantly increased in the hearts of DCM animals compared to WT animals as seen by the increased EPR signal amplitude of CM^∙^ formation (Figures 4(a) and 4(b)). This was further supported by measurements of ROS damage.

Free radical species are highly reactive and can oxidize proteins, lipids, and DNA, which may promote the onset of cardiovascular diseases when ROS accumulate in the heart [29]. Reduced glutathione (GSH) is a vital scavenger of ROS and inhibits lipid peroxidation while also playing a role in detoxification of hydrogen peroxide by glutathione peroxidases [15]. The GSH/GSSG ratio, a marker of oxidative stress and redox status, was significantly decreased in the hearts of DCM animals and cardiomyopathy patients (patient clinical characteristics are provided in Table 1) compared to WT animals and human donors, respectively, signifying increased oxidative stress (Figures 4(c) and 4(f)). Additionally, when protein side chains such as proline, arginine, lysine, and threonine become oxidized, carbonyl groups (aldehydes and ketones) are produced [17]. Compared to WT animals and human donors, carbonyl content was significantly elevated in the hearts of DCM animals and cardiomyopathy patients, respectively, marking increased oxidative stress in the diseased hearts (Figures 4(d) and 4(g)). Lipid peroxidation also serves as an indicator of cellular injury and oxidative stress [30]. Compared to WT animals and human donors, MDA and HAE content were significantly elevated in the hearts of DCM animals and cardiomyopathy patients, respectively, indicating increased oxidative stress (Figures 4(e) and 4(h)). Taken together, these data validate the results of our EPR spectra analysis and reveal increased oxidative stress in the hearts of* MYBPC3*-mutated DCM animals and in the hearts of cardiomyopathy patients.

### 3.4. Altered Mitochondrial Function in Cardiac Tissue from DCM Animals

**Figure 5:**
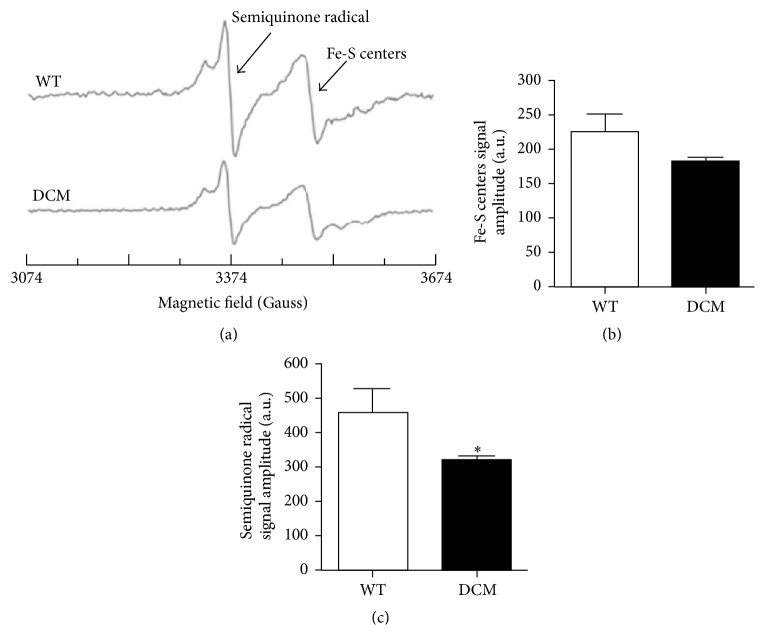
Mitochondrial oxidative stress is augmented in the hearts of DCM animals. (a) Representative EPR spectra of heart tissue homogenates measured at 77 K. Semiquinone free radicals and Fe(III) from Fe-S centers signals are seen at *g* = 2.01 and *g* = 1.94, respectively. EPR signal amplitude was normalized against the weight of the heart and expressed as arbitrary units per gram. (b) Normalized EPR signal amplitude of the Fe-S centers (*n* = 3 hearts). (c) Normalized EPR signal amplitude of the semiquinone radical (*n* = 3 hearts, ^*∗*^ *P* < 0.026). Data are represented as mean ± SEM. Significant differences (*P* < 0.05) between mean values were determined using Student's *t*-test.

Altered redox balance within mitochondria will affect electron transfer in the electron transport chain (ETC) and leads to decreased energy production and increased superoxide generation. Thus, the elevated ROS observed in DCM hearts may affect mitochondrial performance. To investigate mitochondrial performance in WT and DCM animals, we measured both semiquinone radicals and Fe(III) signals in heart tissue. The free radical EPR signal seen at *g* = 2.01 in the heart tissue originates from mitochondrial semiquinone radicals (Figure 5(a)) [31]. The peak at *g* = 1.94 corresponds to Fe(III) from mitochondrial iron-sulfur (Fe-S) centers (Figure 5(a)) [31]. In hearts from DCM mice, the EPR signal corresponding to Fe-S centers was lower as compared to hearts from WT mice (Figure 5(b)). The EPR signal of semiquinone radicals was significantly decreased in the hearts from DCM mice compared to WT mice (Figure 5(c)). Low temperature EPR results demonstrate that the semiquinone radicals and Fe(III) from Fe-S centers were decreased in the hearts of DCM animals, suggesting increased mitochondrial oxidation and reduced ETC efficiency.

## 4. Discussion 

In the present study, we have demonstrated that mutation of the* MYBPC3* gene can result in cardiac oxidative stress, which associates with contractile dysfunction, fibrosis, and myocyte damage in this model of sarcomere protein-mutated cardiomyopathy. Echocardiographic analysis of the hearts from cMyBP-C^(t/t)^ mice at 3 months of age specified elevated LV internal diameter (LVID) and reduced EF and FS (Figure 1) confirming that these mice develop severe DCM and contractile dysfunction as indicated by McConnell et al. [32]. In accordance with these findings, we demonstrate prominent histologic abnormalities in DCM hearts, including eccentric hypertrophy, myocardial disarray, increased cellularity, and a significant elevation in fibrosis compared to hearts from WT animals (Figure 1). Here, we implement forward SHG and FFT analyses of WT and DCM heart tissue to point out clearly disorganized cellular patterning, sarcomere structure, and myocyte damage in DCM hearts (Figures 2 and 3), which is particularly evident near the inner chamber wall in DCM hearts. Furthermore, our backward SHG analyses distinctly indicate elevated levels of collagen in DCM hearts. This finding, in conjunction with our histopathological analysis of Masson's trichrome-stained sections, confirms severe fibrosis in DCM hearts.

The cellular damage observed in DCM hearts produced a complete loss of cellular structure in conjunction with sarcomere disorganization. This is in stark contrast to the studies reported by Plotnikov et al., which, using quantitative SHG, showed disorganized sarcomere patterning in diseased muscles but maintenance of cellular integrity (cell size and morphology) [33]. The discrepancy may be due in part to the differences between disease type and the physiological differences between the skeletal myocytes examined by Plotnikov et al. [33] and the cardiac myocytes used in our analyses. Importantly, it has been suggested that sarcomeric SHG intensity patterns depend greatly upon using fresh tissue sections, which produce a single band when the intensity profile of the SHG signal is along the main myofibril axis [34]. However, the chemical stress that is produced from tissue fixation, elevated ROS due to photoconversion, or external application of hydrogen peroxide (H_2_O_2_) [35], a ROS producer, may cause misalignment of myosin filaments producing the appearance of a double-banded pattern and sarcomere disorganization [34]. In our analysis, despite the evident sarcomere disorganization near the inner chamber regions of DCM hearts, this disorganization is specific to DCM heart tissue and not the fixation process as the sarcomeric structures in similarly fixed WT heart tissue sections displayed a normal pattern.

In genetic cardiomyopathies, mutations within sarcomeric proteins often generate contractile dysfunction within cardiomyocytes producing severe mechanical stress and myocardial remodeling. Therefore, we propose that, over time, progressive deterioration of cardiac pump function perpetuates myocyte and interstitial cell damage or death that may promote increasingly severe oxidative damage that aggravates the disease [36, 37]. Mitochondrial respiration in the form of oxidative phosphorylation is critical for producing sufficient levels of adenosine triphosphate (ATP) to energize cardiac pump function [38]. The perturbation/disruption of mitochondrial respiration results in the partial reduction of oxygen to form the highly reactive oxygen species such as superoxide radicals and hydrogen peroxide [39]. Although, in addition to mitochondrial ROS production, ROS are produced from several intracellular sources including NAD(P)H oxidase, xanthine oxidase, uncoupled nitric oxide synthase, cytochrome c oxidase, and arachidonic acid metabolism [40]. In healthy individuals, the production of ROS is balanced with antioxidant defenses [41]. However, an imbalance between ROS generation and antioxidant defenses leads to oxidative stress [42]. It has been well established that an abundance of ROS is a direct cause of oxidative stress producing lipid peroxidation, DNA and cell membrane damage, and the loss of cells in HF [40, 43, 44]. Indeed, oxidative stress plays a crucial role in the pathophysiology of cardiac remodeling and HF [40]. The implication for oxidative damage and antioxidant therapy in sarcomere protein-mutated cardiomyopathy was previously studied by Marian et al. who tested the effects of antioxidant N-acetylcysteine (NAC), a precursor of glutathione, on diminishing established interstitial fibrosis in a mouse model of human HCM caused by cTnT-Q92 mutation [8]. As the balance between oxidants and antioxidants is important for collagen homeostasis [45], antioxidant therapy may be beneficial in reversing or attenuating established interstitial fibrosis. cTnT-Q92 mice had elevated levels of myocardial MDA and 4-hydroxy-2(E)-nonenal (4HNE), similar to what we observed in cMyBP-C^(t/t)^ DCM heart tissue homogenates (Figure 4(e)), which is indicative of lipid peroxidation and oxidative damage. NAC reduced myocardial concentrations of MDA and 4HNE as well as fibrosis in the hearts of cTnT-Q92 mice [8]. In a follow-up study, Lombardi et al. postulated that treatment of *β*-myosin heavy-chain Q403 transgenic rabbits having established cardiac hypertrophy and preserved systolic function with NAC could reverse cardiac hypertrophy and fibrosis in HCM [9]. Following 12 months of NAC or placebo treatment, transgenic rabbits in the placebo group had a significant elevation in cardiac hypertrophy, fibrosis, and oxidized to total glutathione ratio (GSH/GSSG + GSH) [9]. NAC treatment reversed cardiac and myocyte hypertrophy and interstitial fibrosis, prevented cardiac dysfunction, and restored the oxidized to total glutathione ratio in *β*-myosin heavy-chain Q403 transgenic rabbits [9]. Most recently, using the cTnT^R141W^ transgenic mouse model of DCM, Lu et al. tested whether knockdown of CYP2E1, a cytochrome P450 enzyme that catalyzes the metabolism of toxic substrates, could inhibit oxidative stress and apoptosis of myocytes to prevent DCM development [10]. cTnT^R141W^ mice exhibited increased left ventricular chamber diameter, interstitial fibrosis, swollen mitochondria, and poorly organized myofibrils as well as elevated levels of myocardial MDA and a reduction in GSH, suggesting augmented myocardial oxidative stress [10]. Knockdown of CYP2E1 improved interstitial fibrosis and myofibrillar structure and decreased myocardial oxidative stress and apoptosis in cTnT^R141W^ DCM mice [10]. Similar to these reports, we observed significantly elevated fibrosis (Figures 1(h) and 1(j)), collagen deposition (Figures 2(b) and 2(c)), carbonyl content (Figure 4(d)), and MDA and HAE levels (Figure 4(e)) as well as a reduction in the GSH/GSSG ratio (Figure 4(c)) in cMyBP-C^(t/t)^ DCM hearts and in heart tissue homogenates from cardiomyopathy patients (Figures 4(f)–4(h)), signifying increased myocardial damage and oxidative stress. Importantly, future studies using antioxidant therapy in cMyBP-C^(t/t)^ DCM mice or knockdown/downregulation of ROS generators such as CYP2E1 may lead to a reduction in interstitial fibrosis and myocardial remodeling and could improve cardiac function. This would suggest a therapeutic avenue for the treatment of cardiomyopathies caused by mutation of* MYBPC3* and other contractile proteins.

Previously, to establish direct evidence for increased ROS in failing myocardial tissue, Ide et al. induced HF in adult mongrel dogs by ventricular pacing [46]. EPR spectroscopy was implemented to determine ROS production using freeze-clamped myocardial tissue homogenates that were reacted with the nitroxide radical, 4-hydroxy-2,2,6,6,-tetramethyl-piperidine-*N*-oxyl, followed by detection of spin signals. In HF samples, a significant increase in hydroxyl radicals, as evidenced by the rate of electron spin resonance signal decay, was detected providing direct evidence for enhanced generation of ROS within the failing myocardium [40, 46]. Using this technique, our EPR spectroscopy using spin probe CMH (Figures 4(a) and 4(b)) demonstrated that oxidative stress is increased in the hearts of cMyBP-C^(t/t)^ DCM animals. This result supports our biochemical measurements and the notion that oxidative stress plays a role in cardiac damage and dysfunction in various forms of HF including cardiomyopathy.

As described above, small quantities of mitochondrial ROS are produced in the ETC during mitochondrial respiration, which are typically detoxified by scavenging systems. Previously, Ide et al. demonstrated that inhibiting the ETC at complex I and complex III in normal submitochondrial particles led to a significant elevation in superoxide anion production as measured by EPR spectroscopy with the spin trapping agent 5,5′-dimethyl-1-pyrroline-*N*-oxide [47]. In the presence of NADH, mitochondria from failing canine hearts produced more superoxide anions and were associated with a reduction in complex enzyme activity suggesting mitochondria as an important source of ROS in HF [40, 47]. In addition to their role in cellular respiration, mitochondria are the cellular center for iron homeostasis where iron is used for the biosynthesis of heme and Fe-S clusters [48]. Although, when in excess, labile iron may produce highly reactive oxygen species by the Fenton reaction inducing oxidative damage to cardiomyocytes and myocardial injury [49], thus, regulation of iron homeostasis is critical for cellular viability. Here, EPR spectroscopy revealed a decrease in both semiquinone radicals and Fe(III) from Fe-S centers in DCM animals (Figures 5(a)–5(c)). The decreased EPR signal for semiquinone radicals indicates alterations in mitochondrial respiration [31]. The decreased EPR signal for Fe(III) from Fe-S centers suggests alterations in the iron homeostasis in mitochondria [31]. These results indicate that mitochondrial function is disturbed in the hearts of DCM animals. Together, our EPR studies demonstrate that the impaired mitochondrial respiration and alterations in iron homeostasis are related to increased oxidative stress in the hearts of DCM animals. These data suggest that alteration of normal cardiac mitochondrial function and increased oxidative stress may contribute to the development of cardiomyopathies caused by sarcomere protein mutation.

In this study, we confirm that oxidative stress is elevated at severe HF in a mouse model of sarcomere protein-mutated cardiomyopathy caused by* MYBPC3* mutation. As cMyBP-C is heavily mutated in human cardiomyopathy, determining the mechanisms that contribute to disease progression in cardiomyopathies caused by* MYBPC3* mutations and other sarcomere gene mutations is of critical importance. Currently, medical therapies for HF include suppressing neurohormonal activation and treating fluid volume overload and depressed hemodynamic status. While these pharmacological therapies may improve symptoms and slow the progression of contractile dysfunction, patient prognosis remains poor. Thus, the need for novel therapies directly targeting the factors that perpetuate disease is required to improve cardiac performance and slow or prevent the progression of LV dysfunction and remodeling. Therefore, therapeutic strategies aimed at attenuating oxidative stress could represent a novel treatment avenue to forestall the development of cardiomyopathy in these patients. As such, our future efforts will focus on defining the molecular redox signaling targets for therapeutic intervention to slow the progression of these genetic cardiomyopathies.

## Figures and Tables

**Table 1:** Clinical patient characteristics.

Patients	Sex	Age	Clinical information
1	M	31	NYHA IV, severe ischemia
2	M	43	Unknown
3	M	56	Right ventricular heart failure, Uhl's syndrome, severe right ventricular dysplasia, tricuspid regurgitation, and thin IVS
4	M	54	Severe nonobstructive hypertrophic cardiomyopathy, homozygous TNNT mutant, and triple bypass
5	F	47	Automatic implantable cardioverter-defibrillator, atrial fibrillation, hypothyroid, sinus rhythm, left ventricular hypertrophy, LVEF 20–30%, NYHAII/III, and fractional shortening 21%
6	F	57	Defibrillator, septal hypertrophy, LVEF 15%, and NYHA III
7	F	41	Automatic implantable cardioverter-defibrillator, dual pacemaker, LVEF 50%, and NYHA IV
8	M	61	Atrial fibrillation, LVEF 30%, and NYHA III/IV
9	M	32	HOCM, IVS = 23 mm, and LVOT pressure gradient = 88 mmHg
10	M	60	HOCM, IVS = 23 mm, and LVOT pressure gradient = 77 mmHg
11	F	24	HOCM, IVS = 24 mm, and LVOT pressure gradient = 81 mmHg
12	M	33	HOCM, IVS = 21 mm, and LVOT pressure gradient = 59 mmHg
13	M	17	HOCM
14	M	50	HOCM

Donors	Sex	Age	Cause of death

1	M	56	Hypoxia (hanged)
2	M	45	Unknown
3	M	48	Intracranial hemorrhage, cardiac arrest 23 minutes
4	F	42	Multiple sclerosis, Guillain Barré syndrome
5	F	62	Hypoxia (hanged)
6	F	47	Poland syndrome, hydrocephalus, cardiac arrested 35 min, and celsior cardioplegia
7	M	28	Poland syndrome, hydrocephalus, cardiac arrested 35 min, and celsior cardioplegia
8	M	62	Middle cerebral artery aneurism and hemorrhage, brain herniation
9	F	49	Unknown
10	M	40	Unknown
11	M	29	Unknown
12	M	25	Unknown
13	M	33	Unknown

HOCM = hypertrophic obstructive cardiomyopathy, IVS = interventricular septum, LVEF = left ventricular ejection fraction, LVOT = left ventricular outflow tract, and NYHA = New York Heart Association class.
